# First ovules integument: what roles?

**DOI:** 10.1093/nsr/nwab224

**Published:** 2021-12-11

**Authors:** Brigitte Meyer-Berthaud

**Affiliations:** AMAP, Université de Montpellier, CNRS, CIRAD, INRAE, IRD, France

**Figure 1.**
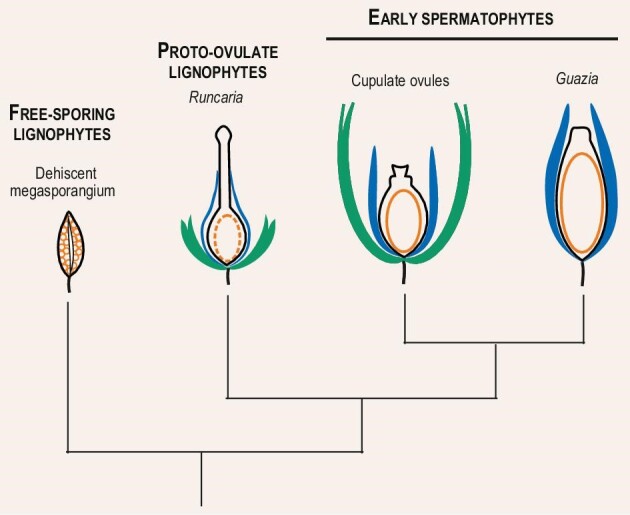
Evolutionary framework for the evolution of Famennian ovules. Megaspores and female gametophytes in orange, megasporangium and nucellus in black, cupule in green, integument in blue.

The Famennian is a period of increased taxonomic diversity for the first representatives of the major plant groups, especially the spermatophytes (i.e. the seed plants) that, today, represent the most abundant and diversified plant group on Earth [1,2]. Early spermatophytes evolved a unique ovule-centered reproductive syndrome that allowed them to germinate and grow rapidly in disturbed habitats and, secondarily, to colonize dry habitats unfavorable to the reproduction of their free-sporing predecessors [3,4] (Fig. 1). In these ovulate organisms, the female gametophyte grows and produces gametes inside the indehiscent megasporangium (nucellus) that differentiates a pollen chamber retaining the pollen grains in its apical part. An integument encloses the nucellus. Like the *Runcaria* protovule that preceded them by 20 million years, Famennian ovules were all surrounded by a cupule, except perhaps *Spermolithus* and *Warsteinia* [5–7]. The latter, however, have been found isolated and may also have had a cupule remaining on the plant that produced them.

The selection pressures that contributed to the evolution of such complex structures are still poorly understood and are the subject of speculation that seems intuitively relevant but has been little tested so far. It is thus widely admitted that the cupule and integument enhanced pollination and protection of the nucellus, but only the former function was investigated using experiments [3,8].

With *Guazia*, Wang *et al*. [9] document the earliest evidence of a naked ovule, i.e. lacking a cupule, a feature that would become widespread in Carboniferous spermatophytes. The authors describe the integument of *Guazia* as consisting of four fused lobes that completely surround the nucellus and form lateral extensions interpreted as wings. *Guazia* would be the second Famennian ovule after *Warsteinia* to have evolved an integument favoring a third function, that of wind dispersal [7].

With specimens up to 20.5 mm long and 5.6 mm wide, the size of *Guazia* exceeds that of all other Famennian ovules recorded so far [3,4,9–11]. Is it not surprising that the most massive ovule of that time evolved wings and was, therefore, the best adapted to wind dispersal, along with the much smaller *Warsteinia?* Were the ‘wings’ of *Guazia*, which are arched inward rather than outward and biconvex in transverse section rather than thin and membranous like those of *Warsteinia*, effective devices for promoting air buoyancy? To answer these questions, tests conducted on the dispersal potential of Permian seeds [12] could be conducted similarly on *Guazia*, perhaps using realistic models produced by 3D printers.

The cupules of early spermatophytes are borne at the tips of fertile fronds (compound leaves), which, when known, differ from the vegetative fronds by the absence of pinnules (photosynthetic appendages). The cupules may have represented the photosynthetic organs of the fertile fronds, contributing to a fourth function, that of the nourishment of the female gametophyte. In *Guazia*, which lacked a cupule, the integument could have taken on this function and been a significant contributor to the development of these large ovules.
